# Dietary and Nutrient Patterns and Brain MRI Biomarkers in Dementia-Free Adults

**DOI:** 10.3390/nu14112345

**Published:** 2022-06-03

**Authors:** Archontoula Drouka, Eirini Mamalaki, Efstratios Karavasilis, Nikolaos Scarmeas, Mary Yannakoulia

**Affiliations:** 1 Department of Nutrition and Dietetics, Harokopio University, 176 71 Athens, Greece; drouka.ar@gmail.com (A.D.); eir.mamalaki@gmail.com (E.M.); myianna@hua.gr (M.Y.); 2 School of Medicine, Democritus University of Thrace, 68100 Alexandroupoli, Greece; stratoskaravasilis@yahoo.gr; 3 1st Department of Neurology, Medical School, National and Kapodistrian University of Athens, Aeginition University Hospital, 115 28 Athens, Greece; 4 The Gertrude H. Sergievsky Center, Department of Neurology, Taub Institute for Research in Alzheimer’s, Disease and the Aging Brain, Columbia University, New York, NY 10027, USA

**Keywords:** dietary patterns, brain MRI, cognitive health, dementia, brain imaging biomarkers

## Abstract

Cognitive impairment is a rapidly growing public health problem. As there is no curative treatment for dementia, the proactive management of modifiable risk factors and the identification of early biomarkers indicative of the cognitive decline are of great importance. Although nutrition is one of the most extensively studied lifestyle factor in relation to cognitive health, its association with brain magnetic resonance imaging (MRI) biomarkers is not well established. In the present work, we review available studies relating dietary or nutrient patterns with brain MRI biomarkers in dementia-free adults. Greater adherence to the Mediterranean diet has been associated with the preservation of structural connectivity and less brain atrophy in adults without dementia. In addition, specific nutrient patterns, characterized by a high intake of antioxidant vitamins, polyphenols and unsaturated fatty acids, have been related to larger brain volume. Although the results are encouraging regarding the role of dietary and nutrient patterns on imaging biomarkers, more well-designed observational longitudinal studies and clinical trials are needed in order to confirm potentially causal relationships and better understand underlying mechanisms.

## 1. Introduction

Aging is associated with cognitive decline leading to mild cognitive impairment and dementia [1,2]. Dementia is a rapidly growing, global, public-health problem. Worldwide, around 50 million people have dementia, and the projected number is estimated to reach 82 million in 2030 and 152 million in 2050 [3]. Dementia is characterized by a progressive reduction in cognitive abilities such as learning, memory, attention, language, intelligence and perception [4,5]. It is noteworthy that the complex pathophysiological process of Alzheimer’s disease (AD), the most common type of dementia, begins many years before the symptoms emerge. Through use of cerebrospinal fluid and imaging biomarkers, AD-related changes can be detected early in cognitively normal older individuals [6]. For example, brain atrophy on structural magnetic resonance imaging (MRI) in a characteristic pattern involving the medial temporal lobes, paralimbic and temporoparietal cortices, has been associated with AD-related neurodegeneration [7,8,9]. Observational longitudinal studies have also suggested that differences in gray matter (GM) can be predictive of conversion of mild cognitive impairment (MCI) to AD [10].

Various environmental factors, such as healthy eating habits, adequate sleep and physical activity, may delay the clinical onset of the disease [11,12,13,14]. Diet is one of the environmental factors that has also been associated with many non-communicable diseases with connections to dementia, such as diabetes and cerebrovascular disease [15]. Numerous studies have indicated the beneficial role of nutrition on the changes in cognitive abilities, the prevalence and the incidence of AD and other dementias [11,15,16,17].

In relation to imaging biomarkers, there are previous reviews on the potential impact of individual nutrients, foods and supplements [18,19,20]; however, the potential intercorrelations of these as depicted in dietary or nutrient patterns and brain MRI have not been collectively reviewed. Dietary and nutrient patterns area more recently used approach in nutrition research, aiming at describing total dietary habits, taking into account the complex interactions between different nutrients or foods [21]. They also provide a useful tool for intervention in clinical practice as they reflect real-life eating behaviors. Thus, the aim of this review was to examine the potential relation between dietary patterns (either a priori or a posteriori) or nutrient patterns that take into account intercorrelations between multiple foods or nutrients, respectively, and imaging biomarkers related to cognitive decline in healthy adults. A priori dietary patterns are based on established hypotheses and guidelines about the role of nutrients in the prevention of diseases; diet is assessed based on adherence to these guidelines, summarized as a score [22]. The Mediterranean diet is one of the most extensively studied a priori dietary patterns. On the other hand, the a posteriori approach is data-driven, and dietary patterns are extracted through statistical methods such as factor or cluster analysis [22].

## 2. Methods

### 2.1. Strategy Used

A search of PubMed and Scopus databases was performed for studies published until February 2022. Search terms were ‘dietary pattern’, ‘nutrient pattern’, ‘MRI’, ‘brain atrophy’ and ‘microbleeds’, used individually and in combination (‘dietary or nutrient pattern and MRI’, ‘dietary or nutrient pattern and/or brain atrophy or microbleeds’). To be included in this review, the studies had to examine the potential association between dietary or nutrient patterns and brain MRI biomarkers, and specifically outcomes such as brain atrophy (through assessment of brain volumes [white matter, grey matter, intracranial] or cortical thickness), cerebrovascular disease, connectivity and functional brain networks. Only studies in humans and in English language were included. Review studies and studies including participants with diagnosis of dementia, depression or schizophrenia were excluded.

### 2.2. Studies Retrieved

The initial database search yielded 510 publications. After screening titles and/or abstracts, the total number was reduced to 370. After careful review, 344 publications were excluded because they did not meet one or more of the inclusion criteria. Almost all of the excluded publications were studies that did not examine a dietary or nutrient pattern but examined individual foods and/or nutrients. The other main reason for exclusion was diagnoses of depression or schizophrenia in the study population. A total of 26 studies met all the criteria.

## 3. Mediterranean Diet

Mediterranean diet (MD) is a term used to describe the traditional eating habits of people living in Crete, South Italy and other Mediterranean regions. This dietary pattern is characterized by an abundance of plant foods: fruits, vegetables, cereals, legumes, nuts and seeds. Olive oil is the principal source of fat. The MD also includes moderate amounts of dairy products (principally cheese and yogurt), low to moderate amounts of fish and poultry, red meat in low amounts; wine is consumed in moderation [23]. Adherence to the MD is evaluated through relevant diet scores: for most of them, a high score indicates high adherence to the MD. MD scores are derived based on two different methodologies; they can be calculated based on population-based cut-offs for specific food groups, such as the Trichopoulou et al. score [24] and variations of it [25], or they can be calculated based on absolute cut-offs for food groups intake according to an apriori description of the traditional MD, such as the Panagiotakos et al. score [26]. The quality of most of these scores, for the assurance of their valid and reliable application, has not been adequately confirmed and needs to be better investigated [27]. Potential associations between the MD and brain MRI derived biomarkers are summarized in Table 1. Participants’ ages were ≥50 years, with the majority of them being female. Only three studies had more male volunteers than female [28,29,30].

### 3.1. Brain Atrophy

The majority of investigations indicated that higher adherence to the MD is positively connected with brain atrophy. Specifically, two studies (one cross-sectional and one clinical trial) found that higher MD adherence was related to larger volumes in anatomical regions such as the hippocampus and the dentate gyrus [28,38]. In addition, higher adherence to the MD was associated with larger brain volumes (total brain volume, grey matter volume and white matter volume) in two other studies (one cross-sectional and one observational longitudinal) [33,39]. Moreover, two cross-sectional studies found that increasing MD adherence was associated with higher cortical thickness, which a sensitive marker of brain atrophy [29,36,37]. It is noteworthy that key regions for AD, namely entorhinal and posterior cingulate cortex, exhibited larger cortical thickness in persons with higher MD adherence [29,36,37]. At the same time, three cross-sectional and one observational longitudinal investigations failed to reveal a significant association between adherence to MD and brain atrophy, expressed either as total brain volume or cortical thickness [30,34,35,40].

Regarding individual MD components, higher fish and walnuts intake and lower meat consumption were associated with larger brain volumes, mean higher cortical thickness and reduced hippocampal occupancy score decline [28,33]. However, there are other studies that do not replicate these associations [39]. The discrepancy in the results may be explained by the different types of foods consumed, for example different types of fish (oily vs low-fat fish) or by the fact that the combination of foods in the context of the MD is more important than individual food groups.

### 3.2. Cerebrovascular Disease

With regards to the white matter lesion load, research so far has produced conflicting results. In particular, the NOMAS and WHICAP studies found that higher adherence to a MD pattern was associated with lower cerebrovascular disease burden, i.e., lower odds of having a MRI infarct [32] and lower white matter hyperintensities (WMH) volume [31]. On the other hand, a recent cross-sectional study with 82 individuals, a sample size with a limited statistical power, did not find an association between MD adherence and WMH [38].

### 3.3. Connectivity

Results from studies examining the potential association between brain connectivity and MD adherence have been mixed. Specifically, adherence to a Mediterranean-style dietary pattern was not related with connectivity of some specific fiber bundles (forceps minor, forceps major, bilateral anterior thalamic radiations, cingulum cingulate gyrus, arcuate, uncinate and inferior longitudinal fasciculi) in the Lothian Birth study [30]. It is worth mentioning that components such as sauces, dressings or puddings, not typical of the traditional MD, were included in the evaluation of this pattern. In contrast, another investigation with a smaller sample and using different methodological approach for analyzing the structural connectivity, showed that higher MD adherence was associated with preserved structural connectivity [35].

### 3.4. Functional Brain Networks

Studies examining the potential association between the MD and functional brain networks were not found.

## 4. Other Dietary or Nutrient Patterns

We found 15 studies describing the potential associations between several dietary and nutrient patterns, either a priori or a posteriori, and brain MRI biomarkers. The majority of studies (8 studies, 9 manuscripts) have a cross-sectional design, 2 have an observational longitudinal design and 4 are intervention studies (Table 2).

### 4.1. Brain Atrophy

Regarding brain atrophy, encouraging results have been reported for healthy dietary and nutrient patterns. Specifically, two cross-sectional studies indicated that higher adherence to healthy diet guidelines, based on national recommendations such as Dutch guidelines or embedded in the Alternative Healthy Eating Index 2010, was associated with larger brain volumes [41,44] and one observational longitudinal study indicated that dietary food diversity, as determined with a quantitative index, based on the proportion of foods that contribute to total energy or the amount of foods and the number of food groups, was connected with larger hippocampal volume [50]. In addition, two studies (one cross-sectional [43] and one observational longitudinal [51]) found mixed results between prudent or western dietary patterns and hippocampal volume. Specifically, Jacka et al. showed that adherence to a healthy dietary pattern was associated with larger hippocampal volume, whereas adherence to a western dietary pattern with smaller hippocampal volume [51]. On the other hand, Zabetian–Targhi et al. did not found any association between prudent, traditional or western dietary patterns and brain atrophy [43].

Interventions promoting the adherence to specific dietary patterns found that a modified, calorie-restricted Mediterranean-DASH Intervention for Neurodegenerative Delay (MIND) diet or a paleolithic-type diet (high intake of lean meat, fish, eggs, fruits, berries, vegetables and nuts, and exclusion of cereals, dairy products, refined fats, sugar and salt) led to increased hippocampus volume [53,54]. It should be noted, that both of them had small sample size (total number of volunteers <40) treated for a short time period, thus caution should be taken when interpreting their findings, as there is a high probability of false-positive results [53,54]. On the other hand, implementing a multidomain intervention, i.e., increasing the adherence to a dietary pattern based on the Finnish Nutrition Recommendations in combination with exercise, cognitive training and vascular risk monitoring, did not result in changes to regional brain volumes or regional cortical thicknesses [52].

When it comes to nutrient, Berti et al. showed that patterns characterized by high intake of vitamins B_12_, D, E, PUFAs, fiber, proteins or monounsaturated fatty acids (MUFAs) were associated with larger total brain volume [49]. On the other hand, higher adherence to patterns based on B vitamins, retinol, proteins, fats or to inflammation-related dietary pattern (characterized by low intakes of calcium, vitamin D, vitamins E, A, B, folate, ω-3 PUFAs, and high intake of cholesterol), were associated with smaller total brain volume [45,46] and gray matter volume in frontal cortex [49].

### 4.2. Cerebrovascular Disease

Higher adherence to healthy eating guidelines, i.e., the Dutch nutrition guidelines and the Finnish Nutrition Recommendations, combined with or without exercise, cognitive training and vascular risk monitoring was not associated with white matter lesion volume, lacunes, or microbleeds [41,52]. Similarly, adherence to a posteriori derived dietary patterns, namely a traditional, a western or a prudent dietary pattern, was not associated with microbleeds, infracts and WMH [43]. Regarding nutrient, two cross-sectional studies have found that higher consumption of flavonoids (total, polymers, flavan-3-ols) or other antioxidants compounds was associated with lower WMH [42,45].

### 4.3. Connectivity

The investigation of the potential associations between connectivity and nutrient patterns revealed that pattern characterized by high intake of PUFAs and vitamin E were associated with higher mean fractional anisotropy [47].

### 4.4. Functional Brain Networks

When it comes to functional brain networks, Zwilling et al. measured nutrient biomarkers in plasma and found that nutrient pattern characterized by ω-3 PUFAs, ω-6 PUFAs or lycopene were associated with enhanced functional brain networks efficiency [48].

## 5. Discussion and Conclusions

Adherence to healthy dietary or nutrient patterns, and especially to the MD, seems to be associated with a more beneficial brain MRI biomarker profile in adults without dementia. Specifically, greater adherence to the MD has been related to preservation of structural connectivity, delayed brain atrophy and less MRI indicators of cerebrovascular disease. In addition, nutrient patterns characterized by high intake of antioxidant vitamins, polyphenols and PUFAs have been related to larger brain volume and lower WMH.

The examination of imaging biomarkers in relation to cognitive decline emerges as a significant area of research; they offer the opportunity to evaluate the progression of AD and measure the efficacy of therapeutics protocols [10]. Thus, they may provide the potential to intervene in an early stage, even before the clinical symptoms of the disease emerge. What is more, the inclusion of brain MRI biomarkers as outcome in an intervention may broaden the therapeutic target and increase the power of interventions aiming to delay cognitive impairment [55].

Previous research has indicated that several dietary components may be beneficially associated with cognitive health and the incidence of dementias [11,14,15,56,57]. The Mediterranean diet is among the most extensively studied dietary pattern in relation to brain MRI biomarkers. Other dietary patterns that include high consumption of fruits, vegetables, olive oil and low consumption of meat, i.e., components characterizing the MD pattern, have also been associated with larger brain volumes, mean thicker cortical thickness and reduced hippocampal occupancy score [28,41,45]. Collectively, these findings suggest that the high intake of specific nutrients, such as unsaturated fatty acids (PUFAs, MUFAs), fibers, vitamin E, vitamin D and flavonoids, may be implicated in the relationships. It should be noted, however, that the mechanisms through which vitamins and unsaturated fatty acids may exert their protective effects may be completely different; vitamins may exert their role through oxidation and effects in total brain atrophy, whereas unsaturated fatty acids through vascular mechanisms [58,59,60]. Further research is needed for the confirmation of the hypotheses and the clarification of the mechanisms.

The limited number of intervention and prospective studies constitutes an important limitation, as there is no adequate evidence for establishing causal relationships. In addition, most epidemiological studies are cross-sectional, and as such they provide a lower level of evidence. There is also considerable heterogeneity in the definition of dietary patterns, either a priori or a posteriori patterns; even in the case of the MD, different scores were used for evaluating the adherence. Similarly, many different brain MRI biomarkers were examined, with different methodologies, a fact that makes an overall interpretation difficult. In addition, the potential impact of lifelong dietary habits (compared with the current dietary or nutrient patterns) has not been evaluated. Finally, the clinical trials identified were relatively of small duration and with small sample size. More large-scale clinical trials are needed to clarify the results.

The study of nutrition, of dietary and nutrient patterns specifically, in relation to brain MRI biomarkers is an emerging area of research. More well-designed observational longitudinal studies and clinical trials studies are needed, in order to better understand potentially causal relationships and underlying mechanisms. Future investigations should also examine aspects that have been rarely explored thus far. For example, the timing of eating, a largely unexplored aspect of the dietary and nutrient patterns, constitutes a promising research field [15]. In addition, analysis of targeted population groups, such as those with unhealthy dietary patterns or other risk factors (genetic, physiological, or lifestyle), might enable the detection of more robust associations between diet and brain imaging biomarkers [15]. As such, dietary modification, based on dietary or nutrient patterns, may be a readily implementable therapeutic intervention, capable of supporting healthy neurocognitive ageing.

## Figures and Tables

**Table 1:** Studies examining the association between higher adherence to the Mediterranean diet and brain MRI biomarkers in adults without dementia.

Study Name	Population Characteristics	Duration	Dietary Assessment or Intervention	MD Assessment Tools	Outcomes				Authors
					Brain Atrophy	CBVD	Connecti-vity	Functional Brain Networks	
Cross-Sectional Studies									
NOMAS	*n* = 966 (59.3% female), ≥55 years old (mean age 72 years old)		FFQ	Trichopoulou MD score		↓ log WMH volume			Gardener et al., 2012 [31]
WHICAP	*n* = 707 (34% male) ≥65 years old (mean age 80.3 years old)		FFQ	Trichopoulou MD score		↓ odds of MRI infarcts, N.S. WMH			Scarmeas et al., 2011 [32]
	*n* = 674 (67% female), ≥65 years old(mean age 80.1 years old)		FFQ	Trichopoulou MD score	↑ TBV, GMV, WMV/Cingulate, parietal, temporal, hippocampus volume and Superior frontal CT distinguish low high MD				Gu et al., 2015 [33]
MCSA	*n* = 672 (52.5% male), 70–89 years old (mean age 79.8 years old)		FFQ	Trichopoulou MD score	increased frontal, average lobar, parietal, and occipital lobe CT				Staubo et al., 2017 [29]
Lothian Birth	*N* = 358 (46.9% female), mean age 79.3 years old		FFQ	PCA (i) Mediterranean-style pattern (ii) Processed pattern	N.S. TBV, GMV, WMV, NAWMV, gFA,		N.S. gMD		Corley et al., 2020 [30]
PIVUS	*n* = 194 (93 female), 75 years old		1 week food diary at 70 years and MRI at 75 years	Trichopoulou MD score	N.S. TBV, WMV, GMV				Titova et al., 2013 [34]
Three-City study Bordeaux	*n* = 146 (58% female), mean age 73 years old		FFQ, 24h diet recall, collected 8.9 years before MRI	Trichopoulou MD score	N.S. GMV, WMV		preserved structural connectivity		Pelletier et al., 2015 [35]
NYU	*n* = 116 (62% female), 30–60 years old (mean age 50 years old)		FFQ	Trichopoulou MD score	↑ CT (+PCC, EC) positively associated with brain structure				Mosconi et al., 2018 [36]
	*n* = 52 (70% female), 25–72 years old (mean age 52 years old)	-	FFQ	Trichopoulou MD score	↑ CT OFC, EC and PCC of the left hemisphere				Mosconi et al., 2014 [37]
UIC	*n* = 82 (52% female), mean age 68.8 years old	-	FFQ	MedDietScore (Panagiotakos)	↑ DG volumes	N.S. WMH			Karstens et al., 2019 [38]
Observational Longitudinal Studies									
Lothian Birth	*n* = 323 70 years old at baseline	10 years (2 MRIs between 3 years)	FFQ	Trichopoulou MD score	↓ TBV reduction N.S. GMV, CT				Luciano et al., 2017 [39]
NYU	*n* = 70 (69% female), 30–60 years old (mean age 49 years old)	3 years	FFQ	Trichopoulou MD score	N.S. CT				Walters et al., 2018 [40]
Randomized Clinical Trials									
DIRECT PLUS	*n* = 224 abdominally obese/dyslipidemic participants (88% male), ≥30 years old (mean age 51 years old)	18 months	(i) Control: healthy dietary guidelines (ii) MD (iii) Green-MD: MD higher in polyphenols (+800 mg/day through green tea) and lower in red/processed meat Both MD groups consumed 440 mg/d polyphenols through walnuts		↓ hippocampal occupancy score decline				Kaplan et al., 2022 [28]

CBVD: cerebrovascular disease, CT: cortical thickness, DG: dentate gyrus, EC: entorhinal cortex, PCC: posterior cingulate cortex, FFQ: food frequency questionnaire, gFA: general factor of fractional anisotropy, g MD: general factor of mean diffusivity, GMV: gray matter volume, MD: Mediterranean diet, MRI: magnetic resonance imaging, MUFA: monounsaturated fatty acids, NAWMV: normal appearing white matter volume, N.S.: not significant, OFC: orbito-frontal cortex, PCA: principal component analysis, PCC: posterior cingulate cortex, SFA: saturated fatty acids, TBV: total brain volume, WMH: white matter hyperintensities, WM: white matter, WMV: white matter volume.

**Table 2:** Studies examining the association between dietary or nutrient patterns other than the Mediterranean diet and brain MRI biomarkers in adults without dementia.

Study Name	Population Characteristics	Duration	Dietary Assessment or Intervention	Outcomes				Authors
				Brain Atrophy	CBVD	Connectivity	Functional Brain Networks	
Cross-Sectional Studies								
The Rotterdam Study	*n* = 4213 (56.8% female), 45–98 years old (mean age 65.7 years old)		FFQ (adherence to Dutch dietary guidelines)	↑ TBV, GMV, WMV, hippocampal volume	N.S. WMLs, lacunes, microbleeds			Croll et al., 2018 [41]
Framingham Heart Study Offspring	*n* = 2086 (53.7% female), (mean age 60.6 years old)		FFQ (flavonoid intake)	N.S. TBV, hippocampal volume	↓ WMH			Shishtar et al., 2020 [42]
Cognition and Diabetes in Older Tasmanians	*n* = 689 (57% male) *n* = 343 T2D, 55–90 years old (mean age 69.9 years old)		FFQ (PCA)					Zabetian–Targhi et al., 2019 [43]
			Prudent DP	N.S. GMV, WMV, hippocampal volumes	N.S. microbleeds			
			Traditional DP	N.S. GMV, WMV, hippocampal volumes	N.S. microbleeds			
			Western DP	N.S. GMV, WMV, hippocampal volumes	N.S. microbleeds			
Whitehall II	*n* = 459 (19.2% female), mean age 59.6 years old		FFQ (AHEI-2010 score)	↑ hippocampal volumes				Akbaraly et al., 2018 [44]
Swedish National study on Aging and Care-Kungsholmen (SNAC-K)	*n* = 417 (59% female), ≥60 years old		FFQ (PCA)					Prinelli et al., 2019 [45]
			DP1: Fiber andAntioxidants	↑ TBV	↓ WMH			
			DP2: LC ω-3 PUFAs andproteins	↑ TBV	N.S. WMH			
			DP3: MUFAs and ω-3,6 PUFAs	↑ TBV	N.S. WMH			
			DP4: SFAs andTrans fat	N.S. TBV	↑ WMH			
			DP5: B- vitamins, retinol, proteins	↓ TBV	N.S. WMH			
WHICAP	*n* = 330 (64% female), mean age 79 years		FFQ					Gu et al., 2018 [46]
			INP	↓ TBV, GMV, WMV				
	*n* = 239 (70% female), ≥65 years old (mean age 84.1 years old)		FFQ (PCA)					Gu et al., 2016 [47]
			DP characterized by ω-3, ω-6, vit. E			↑ FA		
	*n* = 116 (63% female), 65–75 years old (mean age 69 years old)		nutrient biomarker pattern (PCA)					Zwilling et al., 2019 [48]
			ω-6 PUFAs				enhanced functional brain networks efficiency	
			ω-3 PUFAs				enhanced functional brain networks efficiency	
			lycopene				enhanced functional brain networks efficiency	
NYU	*n* = 52 (71% female), mean age 54 years old		FFQ (PCA)					Berti et al., 2015 [49]
			DP1: vitamin B andminerals	N.S. GMV				
			DP2: vitamin E andminerals	↑ GMV				
			DP3: antioxidants andfibers	N.S. GMV				
			DP4: vitamins D andB_12_	↑ GMV				
			DP5: Fats	↓ GMV				
Observational longitudinal studies								
NILS-LSA	*n* = 1683 (50.6% male), 40–89 years old	2 years	3-day weighed dietary records, dietary diversity through QUANTIDD	↓ hippocampal volume N.S. GMV				Otsuka et al., 2021 [50]
PATH	*n* = 255 (46% female), 60–64 years old (mean age 62.6 years old)	4 years	FFQ (PCA)					Jacka et al., 2015 [51]
			Prudent DP	↑ left hippocampal volume				
			Western DP	↓ hippocampal volume				
Randomized clinical trials								
FINGER (multi-domain intervention)	*n* = 112 (59 intervention), 60–77 years old (mean age 70 years old)	2 years	Intervention: diet (based on the Finnish Nutrition Recommendations), exercise, cognitive training, vascular risk monitoring Control: general health advice	N.S. CT, total hippocampal volume total intracranial volume, GMV	N.S. WMLs			Stephen et al., 2019 [52]
	*n* = 37 obese female, 40–60 years old (mean age 49 years old)	3 months	Intervention: calorie-restricted modified MIND diet, Control: calorie-restricted standard control diet	↑ IFG, N.S. cerebellum white matter or cerebellum cortex				Arjmand et al., 2022 [53]
	*n* = 30 with type 2 diabetes, 30–75 years old (female were postmenopausal)	3 months	Paleolithic diet (*n* = 12) with and without high intensity exercise (*n* = 12), control (*n* = 6)	↑ volume of the right posterior hippocampus				Stomby et al., 2017 [54]

AHEI: alternate healthy eating index, CBVD: cerebrovascular disease, CT: cortical thickness, DG: dentate gyrus, DP: dietary pattern, FA: fractional anisotropy, FFQ: food frequency questionnaire, fMRI: functional magnetic resonance imaging, GMV: gray matter volume, IFG: inferior frontal gyrus, INP: inflammation-related nutrient pattern, LC: long chain, MIND: Mediterranean-DASH intervention for neurodegenerative delay, MRI: magnetic resonance imaging, MUFAs: monounsaturated fatty acids, NBPs: nutrient biomarker patterns, N.S.: not significant, PCA: principal component analysis, PUFAs: polyunsaturated fatty acids, QUANTIDD: quantitative index for dietary diversity, rs-fMRI: resting state functional magnetic resonance imaging, SFAs: saturated fatty acids, TBV: total brain volume, WM: white matter, WMH: white matter hyperintensities, WMLs: white matter lesions, WMV: white matter volume.

## Data Availability

No new data were created or analyzed in this study. Data sharing is not applicable to this article.
